# DCE-MRI biomarkers in the clinical evaluation of antiangiogenic and vascular disrupting agents

**DOI:** 10.1038/sj.bjc.6603515

**Published:** 2007-01-09

**Authors:** J P B O'Connor, A Jackson, G J M Parker, G C Jayson

**Affiliations:** 1 Imaging Science and Biomedical Engineering, University of Manchester, Oxford Road, Manchester M13 9PT, UK; 2 Cancer Research UK Department of Medical Oncology, Christie Hospital, Wilmslow Road, Manchester M20 4BX, UK

**Keywords:** angiogenesis inhibitors, biomarkers, clinical trials, dynamic contrast-enhanced magnetic resonance imaging, image analysis

## Abstract

Dynamic contrast-enhanced magnetic resonance imaging (DCE-MRI) is now frequently used in early clinical trial assessment of antiangiogenic and vascular disrupting compounds. Evidence of drug efficacy and dose-dependent response has been demonstrated with some angiogenesis inhibitors. This review highlights the critical issues that influence T_1_-weighted DCE-MRI data acquisition and analysis, identifies important areas for future development and reviews the clinical trial findings to date.

## ANGIOGENESIS: A TARGET FOR ANTICANCER THERAPY

In order to survive and grow beyond a few hundred micrometers, tumours require adequate oxygen and nutrient delivery, as well as removal of waste products. Angiogenesis, the process by which tumours develop a circulatory blood supply, results in the development of vascular networks that are both structurally and functionally abnormal. Compounds that disrupt new vessel formation (antiangiogenic) or destroy existing vessels (vascular disrupting) offer potential targets for novel anticancer therapy (Baluk *et al*, 2005). This strategy has been validated by recent studies of the anti-vascular endothelial growth factor (VEGF) antibody bevacizumab, which have shown improvement in clinical outcome in phase III randomised-controlled trials in colorectal (Hurwitz *et al*, 2004) and other common human cancers.

Angiogenesis inhibitors in clinical development pose challenges for phase I/II trial design. Because they reduce tumour growth or prevent metastases through primarily cytostatic modes of action such as, selectively inhibiting membrane receptors, cell cycle regulators or other signalling pathways, conventional end points based on reduction in tumour size may be inadequate for evaluating clinical response. Alternative imaging biomarkers of angiogenesis are being sought, which can serve as early indicators of drug activity in clinical trials and may facilitate early pharmacodynamic assessments by speeding up the go/no-go decision-making process (Jayson and Waterton, 2005).

Dynamic contrast-enhanced magnetic resonance imaging measurements have been incorporated as biomarkers of drug efficacy in clinical trials of angiogenesis inhibitors. The technique is promising, but its practical application is far from straightforward. The various analysis methods employed have considerable influence on the interpretation of derived parameters and their value as potential biomarkers and/or surrogate end points (Parker and Buckley, 2005). In this review, we outline the requirements of imaging biomarkers for clinical trials of novel agents and highlight relevant features of T_1_-weighted DCE-MRI that should be considered when implementing the technique in human studies. We then assess the clinical findings to date and outline future directions for DCE-MRI in anticancer drug development.

## GENERAL PRINCIPLES OF T_1_-WEIGHTED DCE-MRI IN CLINICAL TRIALS

Dynamic contrast-enhanced magnetic resonance imaging is a noninvasive quantitative method of investigating microvascular structure and function by tracking the pharmacokinetics of injected low-molecular weight contrast agents as they pass through the tumour vasculature. The technique is sensitive to alterations in vascular permeability, extracellular extravascular and vascular volumes, and in blood flow (F). It does not involve ionising radiation, provides good spatial resolution and can be performed on standard specification 1.5 Tesla clinical systems. In this respect, MRI has practical advantages over computed tomography (CT) and positron emission tomography (PET) in evaluating angiogenesis.

**Figure 1:**
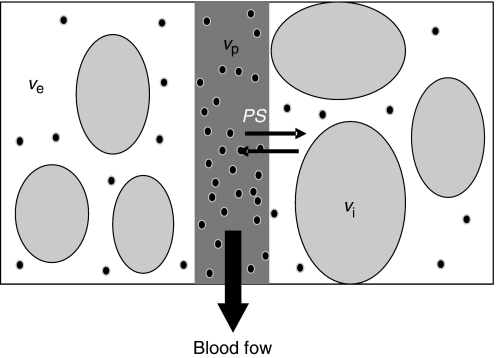
Compartmental modelling of the tumour microvasculature: blood flows through the tumour enabling contrast media molecules (represented as black dots) to distribute in two potential compartments – the blood plasma volume *v*_*p*_ and the volume of the extravascular extracellular space *v*_*e*_. Clinically available MRI contrast agents do not leak into the intracellular space *v*_*i*_. Contrast agent leakage is governed by the concentration difference between the plasma and the extracellular extravascular space and by the permeability and surface area of the capillary endothelia, expressed as *PS*.

In T_1_-weighted DCE-MRI, an intravenous bolus of gadolinium contrast agent enters tumour arterioles, passes through capillary beds and then drains via tumour veins. Gadolinium ions are paramagnetic and interact with nearby hydrogen nuclei to shorten T_1_-relaxation times in local tissue water. This causes increase in signal intensity on T_1_-weighted images to a variable extent within each voxel. The degree of signal enhancement is dependent on physiological and physical factors, including tissue perfusion, arterial input function (AIF) (AIF: the concentration-time course of contrast agent in the artery supplying the vascular bed), capillary surface area, capillary permeability and the volume of the extracellular extravascular leakage space (EES). T_1_-weighted DCE-MRI analysis generates parameters that represent one of, or combinations of these processes, and can be used to measure abnormalities in tumour vessel flow, blood volume, permeability, tortuosity and interstitial pressure (Figure 1). However signal enhancement will also be affected by contrast agent dose, the native T_1_-relaxation time of each tissue and choice of imaging sequences.

Dynamic contrast-enhanced magnetic resonance imaging strategies vary, but, in general, three types of imaging data are acquired. Initial images localise the tumour and provide anatomical information. Next, sequences that allow calculation of baseline tissue T_1_-values before contrast agent administration are acquired to enable subsequent analysis. Finally, dynamic data are acquired every few seconds in T_1_-weighted images over a period of around 5–10 min. Dynamic sequences are subject to innate trade-offs between spatial resolution, temporal resolution (how quickly each image is acquired) and anatomical coverage. Fast T_1_-weighted spoiled gradient echo sequences are generally used as they allow good contrast medium sensitivity, high signal-to-noise ratio, adequate anatomical coverage and rapid data acquisition (Parker and Buckley, 2005).

## IMPORTANT CONSIDERATIONS FOR IMAGE ACQUISTION AND ANALYSIS

Comprehensive discussion of the technical aspects of DCE-MRI image acquisition and analysis is beyond the scope of this paper. However, some factors are briefly considered as selection of MRI sequences and data analysis methods determine not only the range of parameters available, but also their precise meaning.

### Analysis: descriptive or physiological?

Several analysis methods can be applied to DCE-MRI data. Features of the signal intensity–time curve (e.g. gradient, overall shape, time to 90% maximum enhancement) represent simple descriptions of contrast agent distribution. However, these measures show considerable variation between acquisition method and individual examinations, making direct comparison between patients and trials difficult. Conversion of signal intensity into contrast agent concentration data allows more robust analysis of contrast agent kinetics. However, unlike dynamic CT or PET, the relationship between signal intensity and contrast agent concentration is not linear, making conversion of the signal intensity data far from straightforward (Tofts *et al*, 1999; Parker and Buckley, 2005).

Parameters that describe the shape of the contrast agent concentration–time curve represent a combination of flow, blood volume, vessel permeability and EES volume. One such quantity, the initial area under the contrast agent concentration–time curve (IAUC) is easy to calculate (model-free), reasonably reproducible and is routinely used as a biomarker in drugs trials. However, IAUC has a complicated and incompletely defined relationship with underlying tumour physiology and represents a composite of physiological processes (Tofts *et al*, 1999).

### Which model should be used?

Pharmacokinetic models can be applied to contrast agent concentration data to enable estimates of physiological characteristics such as flow and capillary endothelial permeability. Modelled parameters are in theory more ‘physiologically meaningful’ than simple descriptors, such as IAUC, and are independent of acquisition protocol and solely reflect tissue characteristics. Thus, they are suitable measurements for multicentre studies with variation in image acquisition protocols and equipments (Leach *et al*, 2005).

Consensus opinion recommends that simple models describing the volume transfer coefficient of contrast between the blood plasma and the EES (*K*^trans^) and the size of the EES (*v*_e_) should be used along with IAUC in assessing antiangiogenic and vascular disrupting agents in clinical trials (Leach *et al*, 2005). Other related measures such as the rate constant (*k*_ep_), which describes the ratio of *K*^trans^/*v*_e_ have also been used. Several models have been applied to clinical trial data to enable calculation of *K*^trans^ and *v*_e_, many of which are equivalent (Larsson *et al*, 1990; Tofts and Kermode, 1991).

Changes in *F*, endothelial permeability and endothelial surface area produce changes in measurements of *K*^trans^ (or an equivalent parameter, such as *K*_i_) in these models, and the specific contribution of the individual components cannot be identified. Importantly, the interpretation of *K*^trans^ varies depending on the relationship between *F* and capillary permeability–surface area product (*PS*). When tissue contrast delivery is ample (*F*≫*PS*) *K*^trans^ represents the *PS* per unit volume of tissue, for *trans*-endothelial transport between plasma and EES (*K*^trans^∼*PS*). In limited perfusion (*PS*≫*F*) *K*^trans^ represents the *F* per unit volume of tissue (*K*^trans^∼*F*) (Tofts *et al*, 1999). In these simple models, both *K*^trans^ and *v*_e_ calculation are relatively stable but lack physiological specificity.

Extensions of this model (Tofts 1997) are more complex, but enable calculation of blood plasma volume (*v*_p_) and provide more accurate estimations of *K*^trans^ and *v*_e_. More comprehensive models allow direct quantification of flow (*F*), extraction fraction (*E*), *v*_e_ and mean capillary transit time (*τ*) (St Lawrence and Lee, 1998). Here, rather than defining the composite parameter *K*^trans^, it is possible to separate *F* and *PS*. However successful application of this model requires a temporal resolution in the order of 1 s to measure *τ* accurately, which limits its application in clinical trials (Jayson *et al*, 2005; Parker and Buckley, 2005).

*K*^trans^ does not purely measure capillary permeability in any of these models (although it is often assumed to do so). Instead, its exact meaning depends on the kinetic model used for analysis. Changes in *K*^trans^ may also represent different physiological processes in different individuals within the same patient cohort (e.g. reduction in *K*^trans^ could represent reduced flow, reduced permeability or a combination of the two). The choice of analysis techniques is therefore not straightforward and reflects a compromise between parameters that are either relatively simple but poorly specific or physiologically congruent but less stable.

### Imaging protocol

Incorporating DCE-MRI into clinical trials has required considerable technical expertise from basic scientists and clinicians in preparing nonstandard MRI sequences and in-house software for data analysis. Hence, early DCE-MRI studies varied in data acquisition and analysis methods, making comparison difficult and confusing. Multicentre trials require uniform image acquisition and analysis favouring reproducible machine-independent protocols. Such studies require careful quality control and are increasingly managed by contract research organisations specialising in advanced imaging applications. Multicentre DCE-MRI trial feasibility has been demonstrated by studies of the tyrosine kinase inhibitors AG-013736 (Liu *et al*, 2005) and BIBF 1120 (Padhani *et al*, 2006). At present, two baseline scans are recommended for all studies (to define parameter reproducibility) (Leach *et al*, 2005), but guidelines on the timing of MRI scanning and the choice of imaging parameters are less clear.

### Importance of the AIF

In theory, the models described above require direct measurement of an AIF along with the tumour contrast agent concentration–time course curve. These two functions are then used to quantify the passage of contrast agent through the tumour. Ideally, the AIF should be measured for each examination, as it varies between individuals and visits reflecting physiological variation in cardiac output, vascular tone, renal function and injection timing. Unfortunately, AIF measurement is technically demanding and, at best, produces an indirect measurement from a nearby large artery that may differ from the vessel supplying the tumour. Therefore, many groups use an idealised mathematical function instead, which allows far greater freedom in the imaging protocol by relaxing requirements on temporal resolution, slice positioning and sequence choice, but makes no attempt to reflect the true blood supply to the tumour at each examination (Parker and Buckley, 2005). Whichever technique is used, AIF measurement has a major impact on data analysis and clinical results – inaccuracy in the form or scale of the AIF affects the magnitude of all of the modelled parameters and their reproducibility.

### Region of interest and statistical analysis

Data analysis is performed on a defined region of interest (ROI) that encompasses all or part of the tumour. A single average-enhancement curve can be extracted and used to generate values of parameters of interest (such as IAUC or *K*^trans^), and the same parameters can then be compared following therapy (Dowlati *et al*, 2002; Morgan *et al*, 2003; Liu *et al*, 2005; Mross *et al*, 2005a; Thomas *et al*, 2005). This method ignores heterogeneity within the tumour. Alternatively, data can be extracted from each voxel within the ROI and summary statistics such as the mean and s.d., or median and interquartile range may be calculated (Jayson *et al*, 2002, 2005; Galbraith *et al*, 2003; Evelhoch *et al*, 2004). This second method can describe both normal and nonnormal data distributions and provides limited information regarding microvascular heterogeneity. In practice, both methods have been used in trials of antiangiogenic and vascular disrupting agents.

### Data quality

Established and agreed policies for quality control are essential. T_1_-values should be checked against a reference range for the relevant magnetic field strength. Significant motion artefact, AIF and ROI definition and signal-to-noise ratio should be assessed and corrected. If correction is not possible, then corrupted datasets should be removed from subsequent analysis (Buonaccorsi *et al*, 2006). These considerations are important because they influence the accuracy of data (hence ensure quality control) and the significance/power of results (hence avoid dataset exclusion). In practice data are frequently suboptimal and can lead to trial data being excluded or of limited value (Eder *et al*, 2002; Morgan *et al*, 2003; Stevenson *et al*, 2003; Liu *et al*, 2005; O'Donnell *et al*, 2005).

## CLINICAL TRIALS TO DATE: METHODS AND RESULTS

At the time of writing, 21 trials of antiangiogenic compounds (Eder *et al*, 2002; Jayson *et al*, 2002, 2005; Morgan *et al*, 2003; Thomas *et al*, 2003, 2005; Conrad *et al*, 2004; Medved *et al*, 2004; Overmoyer *et al*, 2004; Xiong *et al*, 2004; Drevs *et al*, 2005; Liu *et al*, 2005; Mross *et al*, 2005a, 2005b; O'Donnell *et al*, 2005; O'Dwyer *et al*, 2005; Rosen *et al*, 2005, 2006; Padhani *et al*, 2006; Watson *et al*, 2006; Wedam *et al*, 2006) and six trials of vascular disrupting compounds (Dowlati *et al*, 2002; Galbraith *et al*, 2002, 2003; Stevenson *et al*, 2003; Evelhoch *et al*, 2004; McKeage *et al*, 2006) have employed T_1_-weighted DCE-MRI analysis and described their protocols and findings in enough detail to allow critical appraisal (Tables 1 and 2). Most were small cohort single-centre phase I trials in patients with advanced solid tumours, although a small number of phase II trials have incorporated DCE-MRI (Wedam *et al*, 2006). Marked variation in tumour size (Evelhoch *et al*, 2004), anatomy and pathophysiology and previous treatment (Morgan *et al*, 2003) have made data evaluation difficult and may have masked subtle drug effects, prompting a move towards stricter inclusion criteria (Morgan *et al*, 2003; Mross *et al*, 2005a; O'Dwyer *et al*, 2005) or using an intra-patient dose escalation design (Jayson *et al*, 2002; Stevenson *et al*, 2003).

### Biomarker evidence of drug effect – what does it mean?

Dynamic contrast-enhanced magnetic resonance imaging parameters are subject to random error, biological variation and systemic inaccuracies that cause day-to-day variation in measured values. Many investigators consider a change in *K*^trans^ of ⩾40% as likely to represent a true difference in the parameter as some evidence suggests it correlates with disease stability/response, but in practice the confidence interval for each parameter depends on the choice of model, AIF methods and ROI definition (Roberts *et al*, 2006). Knowing the intrapatient variability for the study population is essential to have confidence that a parameter change is due to drug effect. Therefore, many (though not all) centres perform two baseline scans to measure reproducibility for each trial dataset, in accordance with published guidelines (Galbraith *et al*, 2003; Jayson *et al*, 2005; Padhani *et al*, 2006).

Evidence of drug efficacy has been demonstrated with DCE-MR1 in several trials of antiangiogenic drugs. Significant reductions in *K*^trans^ have been reported in patients with advanced breast cancer receiving bevacizumab alone, implying reduction in *F* and/or permeability. *K*^trans^ reduction was increased following a further six cycles of bevacizumab with conventional chemotherapy. However changes in *K*^trans^ did not predict response rate (RR) (Wedam *et al*, 2006). Several tyrosine kinase inhibitors, notably AG-013736 (Liu *et al*, 2005), BIBF1120 (Mross *et al*, 2005b) and AZD2171 (Drevs *et al*, 2005) have all shown dose-dependent reductions in *K*^trans^ and IAUC without demonstrating clinical response.

Few trials have demonstrated a relationship between DCE-MRI biomarker and clinical outcome measure. Correlation of *K*^trans^ reduction and RR and progression-free survival (PFS) has been shown with BAY 43-9006 (O'Dwyer *et al*, 2005). Reduction, in *K*_i_ of ⩾40% has predicted which patients progressed with glioblastoma multiforme in a trial of PTK787/ZK222584 (PTK/ZK) (Conrad *et al*, 2004). Changes in *K*^trans^ following trials of AZD2171 (Drevs *et al*, 2005) and BMS-582664 (Rosen *et al*, 2006) have helped define the effective dose to take into phase II studies.

The studies outlined above show that DCE-MRI biomarkers can provide early indicators of efficacy, dose and outcome. However, such findings do not guarantee success in phase III development. Three related trials of PTK/ZK reported promising early results with DCE-MRI biomarkers. Patients with colorectal carcinoma liver metastases (mixture of disease stage and extent of prior therapy) showed a dose-dependent reduction in *K*_i_ of 43% at day 2, which was greatest in higher-dose groups (⩾1000 mg once daily), where *K*_i_ was reduced by 58% on day 2 and 53% at the end of cycle 1 (EC1). Reduction in tumour enhancement at day 2 and EC1 (measured by reduction in *K*_i_ from baseline) predicted disease progression and was positively correlated with reduction in tumour size (Morgan *et al*, 2003). Statistically significant dose-dependent changes in *K*_i_ from baseline at day 2 and day 28 were identified in two subsequent studies of PTK/ZK in patients with mixed solid tumours (⩾1000 mg once daily) (Mross *et al*, 2005a; Thomas *et al*, 2005). A biologically active dose of 1000 mg was identified in all three studies.

Two subsequent randomised phase III trials of PTK/ZK with standard treatment in patients with metastatic colorectal carcinoma suggest that DCE-MRI parameter changes may be necessary but not sufficient biomarkers of drug efficacy. Oral PTK/ZK (1250 mg q.d.s) or placebo were administered with oxaloplatin/5-flurouracil/leucovorin (FOLFOX4) as first-line (CONFIRM-1) (Hecht *et al*, 2005) or second-line therapy (CONFIRM-2) (Koehne *et al*, 2006). Analysis of PFS in CONFIRM-1 did not achieve statistical significance. Overall survival (OS) data are awaited. Response rate between trial arms were not significantly different and the primary endpoint of OS was not met in CONFIRM-2, although PFS was significantly longer in the PTK/ZK arm (5.5 *vs* 4.1 months; hazard ratio 0.83; *P*=0.026).

Fewer trials of vascular disrupting agents have incorporated DCE-MRI biomarkers. Evidence of drug efficacy has been shown in the flavonoid DMXAA (Galbraith *et al*, 2002), the antitubulin agent CA-4-P (Galbraith *et al*, 2003) and the colchicine analogue ZD6126 (Evelhoch *et al*, 2004). Evaluation of CA-4-P helped define the biologically active and maximum tolerated doses.

### Negative results – how confident are we?

Three factors are imperative if DCE-MRI biomarkers are to detect true negative results and hence increase the value of DCE-MRI in evaluating antiangiogenic and vascular disrupting agents. Firstly, correct scan schedule is required. For example, vascular disrupting agent drug effects are typically seen within hours of administration and may be lost within 24 h. Thus, DCE-MRI trials of vascular disrupting agents require imaging at baseline, 4–6 and at 24 h. In contrast, antiangiogenic drug effects typically occur within days to weeks and may persist for weeks to months, so that imaging must be performed at these time points.

Secondly, correct and robust parameters must be selected for each compound and used appropriately. Most studies employ changes in mean or median IAUC and *K*^trans^ irrespective of the proposed drug mechanism. Although changes in *K*^trans^ may be meaningful in trials of VEGF/VEGF-receptor inhibitors, its usefulness in evaluating other compounds is less clear. Parameters, such as change in relative enhancing fraction within the total tumour volume, influenced by altered interstitial pressure, may be more informative in assessment of platelet-derived growth factor/platelet-derived growth factor receptor inhibitors (Jayson *et al*, 2005). Parameter changes require correct interpretation, as for example *K*^trans^ changes may reflect altered flow or vascular permeability. Data quality (minimal motion, well-defined AIF and reproducible analysis) must be sufficient to allow measured parameters changes to be detected.

Finally, data analysis is crucial and must be critically appraised in each trial. Current methods that produce summary parameters oversimplify data and may mask critical information concerning tumour heterogeneity. Alternative methods of data evaluation, such as histogram analysis (Watson *et al*, 2006) may have a role in demonstrating changes in heterogeneity. Equally, tumour subregions may require separate analysis in order to detect subtle drug effects (e.g. rim-core differential of vascular disrupting agent action) (Walker-Samuel *et al*, 2006), which may otherwise be obscured and be reported as false negative results.

## CONCLUSIONS AND FUTURE CHALLENGES

Magnetic resonance imaging biomarkers of tumour angiogenesis require validation, ideally against clinical outcome measures such as OS, PFS or RR. At present, an insufficient number of clinical studies have correlated changes in *K*^trans^, IAUC or other DCE-MRI biomarkers with clinical outcome to allow their adoption as established surrogate end points of drug effects. Validation against histopathology biomarkers such as microvascular density is problematic in DCE-MRI, where micrometer scale biopsy changes must be compared against voxel resolution in millimetres. Nonetheless, histopathology validation is important and can substantiate the use of a biomarker in phase I/II trials. Both animal models and clinical studies are likely to be required to achieve comprehensive validation (Jayson and Waterton, 2005; Leach *et al*, 2005).

Dynamic contrast-enhanced magnetic resonance imaging is considerably more complex than conventional anatomical imaging. Acquisition and analysis protocols place a significant demand on both patients and staff. However, initial studies show that the technique is feasible, and T_1_-weighted DCE-MRI parameters have provided considerable noninvasive information concerning tumour biology and response to treatment. Future developments in image acquisition and analysis are evolving rapidly and may further increase the potential that DCE-MRI has to play in future assessment, diagnosis and follow-up of cancer.

## Figures and Tables

**Table 1:** Antiangiogenic agents evaluated by DCE-MRI in clinical trials

**Study**	**Agent**	**N**	**Tumour group^a^**	**DCE-MRI biomarker**	**Evidence of drug effect**	**Inform dose**	**Predict outcome**
*Anti-VEGF antibody*							
Jayson	HuMV833	20	Mixed	*K*^trans^, *k*_*ep*_, *rBV*	Reduction in *K*_ep_ but no dose relationship	No	No
Overmoyer	Bevacizumab	26	Breast	*k*_ep_	Reduction in *K*_ep_	—	—
Wedam	Bevacizumab	20	Breast	*K*^trans^, *v*_*e*_	↓ *K*^trans^ 34% EC1 (BV alone) & 75% EC7 (BV+ cytotoxic)	No dose relationship	*K*^trans^ did not predict RR
							
*Tyrosine kinase inhibitor*							
Conrad	PTK/ZK	14	GBM	*K*_i_, *rBV*	Dose-dependent ↓ *K*_i_ ∼40% at d28	—	*K*_i_ predicts progression
Morgan	PTK/ZK	26	CRC	*K*_i_	Dose-dependent ↓ *K*_i_ – 58% in ⩾ 1000 mg at d2, 53% at d28	Optimum ⩾1000 mg	*K*_i_ predicts RR & progression
Mross	PTK/ZK	27	CRC/Breast	*K*_i_	Dose-dependent ↓ *K*_i_ – 54% in ⩾1000 mg at d2, 51% at d28	Optimum ⩾1000 mg	*K*_i_ predicts RR & progression
Thomas	PTK/ZK	35	Mixed	*K*_i_	Dose-dependent ↓ *K*_i_ – 46% in ⩾1000 mg at d2, 40% at d28	Optimum ⩾1000 mg	*K*_i_ predicts RR & progression
Drevs	AZD2171	24	Mixed	IAUC	↓ IAUC of ⩾40% in five out of seven patients	Effective 20–45 mg	—
Medved	SU 5146	19	Mixed	IAUC	Progressive disease despite ↓ IAUC	—	—
O'Donnell	SU 5146	24	Mixed	*K*^trans^, *v*_*e*_	No consistent relationship with clinical measures	No dose relationship	—
Liu	AG-013736	17	Mixed	IAUC, *K*^trans^	Dose-dependent ↓ IAUC, *K*^trans^ d2 and d28. Effect lost d56	—	—
O'Dwyer	BAY 43-9006	12	Renal CC	*K*^trans^, *v*_*e*_	Mean reduction *K*^trans^ 61%		*K*^trans^ predicts RR & PFS
Rosen	AMG-706	18	Mixed	IAUC	↓ IAUC of ⩽61%	—	—
Rosen	BMS-582664	7	Mixed	*K* ^trans^	↓ *K*^trans^ ⩾40% in 600–800 mg cohort	Effective 600–800 mg	—
Xiong	SU6668	4	Mixed	IAUC, slope	None	No	No
Mross	BIBF1120	27	Mixed	IAUC, *K*_i_	Reduced IAUC & *K*_i_ ⩾40% in higher dose groups	No	—
Padhani	BIBF1120	35	Mixed	IAUC, *K*^trans^, *k*_*ep*_	No consistent relationship with clinical measures	No	—
							
*Anti-PDGFR-β antibody Fab'*							
Jayson	CDP860	8	CRC/Ovary	IAUC, *K*^trans^, *v*_*e*_, *v*_*p*_	↑ vascularised tumour volume in some patients	No	No
							
*Recombinant human endostatin*							
Eder	rhEndostatin	10	Mixed	*K*^trans^, *v*_*e*_	None	No	No correlation with PFS or OS
Thomas	rhEndostatin	21	Mixed	No detail provided	None	No	No correlation with TTP
							
*Anti-αv integrin*							
Watson	CNTO95	22	Mixed	IAUC, *K*^trans^, *v*_*e*,_ *v*_*p*_	No consistent relationship with clinical measures	No	—

CRC=colorectal; d=day; DCE-MRI=Dynamic contrast-enhanced magnetic resonance imaging; EC=end cycle; GBM=glioblastoma multiforme; IAUC=initial area under the contrast agent concentration–time curve; *K_i_* =uni-directional influx constant; *k_ep_*=rate constant; *K*^trans^=bi-directional transfer co-efficient; OS=overall survival; PDGFR=platelet-derived growth factor receptor; PFS=progression-free survival; *rBV*=regional blood volume; Renal CC=renal cell carcinoma; RR=response rate; slope=slope of the uptake curve; TTP=time to progression; VEGF=vascular endothelial growth factor; *v_e_*=volume of the EES; *v_p_*=blood plasma volume

a Tumours were either mixed solid group, or breast, glioblastoma multiforme (GBM), colorectal (CRC), renal cell carcinoma (Renal CC) or epithelial ovarian (Ovary).

**Table 2:** Vascular disrupting agents evaluated by DCE-MRI in clinical trials

**Study**	**Agent**	N	**Tumour^a^ group**	**DCE-MRI biomarkers**	**Evidence of drug effect**	**Inform dose**	**Predict outcome**
*Flavonoid*							
Galbraith	DMXAA	16	Mixed	IAUC, slope, E	Reduction IAUC at 24 h and after last dose	No	No
McKeage	DMXAA	15	Mixed	IAUC, *K*^trans^, *k*_*ep*,_ *v*_*e*_	↑ *v*_*e*_ in 6 pts. No change in IAUC, *K*^trans^, *v*_*p*_	No	No
							
*Anti-tubulin combretastatin analogue*							
Dowlati	CA-4-P	7	Mixed	SI measures	None	No	No
Galbraith	CA-4-P	18	Mixed	IAUC, *K*^trans^, *v*_*e*,_	Dose dependent ↓ *K*^trans^ in 6/16 pts with 52 mg m^−2^ at 4 h	BAD ⩾52 mg m^−2^ and MTD 68 mg m^−2^	No
Stevenson	CA-4-P	10	Mixed	*K*^trans^, *v*_*e*_	No consistent relationship with clinical measures	No	No
							
*Colchicine analogue*							
Evelhoch	ZD6126	9	Mixed	IAUC	dose–dependent reduction 36–72% of IAUC in 6/9 pts at 6 h	BAD ⩾80 mg m^−2^	No

BAD=biologically active dose; d=day; DCE-MRI=Dynamic contrast-enhanced magnetic resonance imaging; E=signal intensity enhancement; hr=hour; IAUC=initial area under the contrast agent concentration–time curve; *K*^trans^=bi-directional transfer co-efficient; *k_ep_*=rate constant; MTD=maximum tolerated dose; pts=patients; SI=signal intensity; slope=slope of the uptake curve; *v_e_*=volume of the EES.

a Tumours were all mixed solid group.
